# Skin Cancer-Associated *S. aureus* Strains Can Induce DNA Damage in Human Keratinocytes by Downregulating DNA Repair and Promoting Oxidative Stress

**DOI:** 10.3390/cancers14092143

**Published:** 2022-04-25

**Authors:** Annika Krueger, Ahmed Mohamed, Cathryn M. Kolka, Thomas Stoll, Julian Zaugg, Richard Linedale, Mark Morrison, H. Peter Soyer, Philip Hugenholtz, Ian H. Frazer, Michelle M. Hill

**Affiliations:** 1 The University of Queensland Diamantina Institute, Faculty of Medicine, The University of Queensland, Translational Research Institute, Woolloongabba, QLD 4102, Australia; a.krueger@uq.edu.au (A.K.); richard.linedale@tri.edu.au (R.L.); m.morrison1@uq.edu.au (M.M.); i.frazer@uq.edu.au (I.H.F.); 2 QIMR Berghofer Medical Research Institute, Herston, Brisbane, QLD 4006, Australia; mohamed.a@wehi.edu.au (A.M.); cathryn.kolka@qimrberghofer.edu.au (C.M.K.); thomas.stoll@qimrberghofer.edu.au (T.S.); 3 Australian Centre for Ecogenomics, School of Chemistry and Molecular Biosciences, The University of Queensland, St Lucia, QLD 4072, Australia; j.zaugg@uq.edu.au (J.Z.); p.hugenholtz@uq.edu.au (P.H.); 4 Dermatology Research Centre, The University of Queensland Diamantina Institute, The University of Queensland, Brisbane, QLD 4102, Australia; p.soyer@uq.edu.au; 5 Dermatology Department, Princess Alexandra Hospital, Brisbane, QLD 4102, Australia; 6 The University of Queensland Centre for Clinical Research, Faculty of Medicine, The University of Queensland, Herston, QLD 4006, Australia

**Keywords:** squamous cell carcinoma, actinic keratosis, *Staphylococcus aureus*, keratinocytes, transcriptomics, proteomics, cancer-promoting toxins, DNA repair, oxidative stress, genotoxicity, phenol soluble modulins

## Abstract

**Simple Summary:**

Squamous cell carcinoma of the skin and precancerous skin lesions, called actinic keratoses, on severely photodamaged skin are often colonized with unusually high amounts of the bacterium *Staphylococcus aureus*. It is not yet known whether this bacterium directly contributes to cancer formation in the skin. To test this, we exposed healthy human skin cells in culture to secreted compounds from *S. aureus* taken from precancerous and cancerous skin lesions. We then determined if the *S. aureus*-treated skin cells start showing typical signs of cancer transformation by looking at the changes in RNA transcripts and proteins. We found that the secreted compounds from some *S. aureus* strains were able to induce cancer-promoting changes in skin cells. Our findings suggest that *S. aureus* colonization on precancerous skin may contribute to cancer development and that there might be benefits of eradicating *S. aureus* on heavily photodamaged skin.

**Abstract:**

Actinic keratosis (AK) is a premalignant lesion, common on severely photodamaged skin, that can progress over time to cutaneous squamous cell carcinoma (SCC). A high bacterial load of *Staphylococcus aureus* is associated with AK and SCC, but it is unknown whether this has a direct impact on skin cancer development. To determine whether *S. aureus* can have cancer-promoting effects on skin cells, we performed RNA sequencing and shotgun proteomics on primary human keratinocytes after challenge with sterile culture supernatant (‘secretome’) from four *S. aureus* clinical strains isolated from AK and SCC. Secretomes of two of the *S. aureus* strains induced keratinocytes to overexpress biomarkers associated with skin carcinogenesis and upregulated the expression of enzymes linked to reduced skin barrier function. Further, these strains induced oxidative stress markers and all secretomes downregulated DNA repair mechanisms. Subsequent experiments on an expanded set of lesion-associated *S. aureus* strains confirmed that exposure to their secretomes led to increased oxidative stress and DNA damage in primary human keratinocytes. A significant correlation between the concentration of *S. aureus* phenol soluble modulin toxins in secretome and the secretome-induced level of oxidative stress and genotoxicity in keratinocytes was observed. Taken together, these data demonstrate that secreted compounds from lesion-associated clinical isolates of *S. aureus* can have cancer-promoting effects in keratinocytes that may be relevant to skin oncogenesis.

## 1. Introduction

Many cancer types present with a distinct microbiome composition and changes in the microbiome can influence disease progression and the response to cancer therapy [1,2,3]. Microbial metabolites, structural components and secreted factors can promote eukaryotic cell proliferation and mutagenesis [3,4]. For example, bacterial species such as *Helicobacter pylori*, *Salmonella typhi* and *Escherichia coli* each have carcinogenic potential due to their ability to secrete products that facilitate oxidative stress and/or DNA damage [5,6,7].

Keratinocyte skin cancers, including basal cell carcinoma, intraepidermal carcinoma (IEC), and squamous cell carcinoma (SCC), as well as their precursors lesion actinic keratosis (AK), are associated with microbial dysbiosis and an increased abundance of the pathogenic bacterium *Staphylococcus aureus* [8,9,10,11,12]. However, it is not clear whether these microbial changes are relevant to keratinocyte cancer development. To date, only two studies have addressed potential mechanisms by which *S. aureus* may drive skin carcinogenesis. Madhusudhan and colleagues [11] reported that the *S. aureus* load in SCC tumour samples significantly correlated with the mRNA expression of human β-defensin-2 in these tissues. Further, they showed that in vitro, *S. aureus* infection induces human β-defensin-2 and has a growth-promoting effect in human SCC cell lines [11]. A second more recent study conducted in our laboratory demonstrated that secreted toxins from AK, IEC and SCC-derived *S. aureus* can cause human keratinocytes to overexpress inflammatory mediators and growth factors associated with skin carcinogenesis [13]. These effects were strain-specific with some isolates being strong inducers of inflammatory markers while others from similar lesions of different patients had no or low proinflammatory activity. A significant positive correlation was observed between inflammatory response and the concentration of particular toxins in the secretome including alpha toxin and phenol soluble modulin (PSM) toxins [13].

In the present study, we have explored potential pro-tumorigenic mechanisms of the *S. aureus* secretome in human skin cells, applying RNA-Seq and shotgun proteomics to primary human keratinocytes after exposure to secretomes derived from AK and SCC-associated *S. aureus*. This exploratory approach has revealed that *S. aureus*-derived compounds can induce SCC biomarkers, downregulate DNA repair pathways, and activate oxidative stress and DNA damage pathways. These omics findings were validated through reactive oxygen species (ROS) and DNA damage measurements in primary keratinocytes following exposure to additional *S. aureus* secretomes. Our findings shed light on the cancer-promoting properties of *S. aureus*, a previously overlooked potential driver of cutaneous SCC development and progression.

## 2. Materials and Methods

### 2.1. Clinical Sampling of Skin Cancer and Precancerous Lesions

Skin swab samples were collected from AK, IEC and/or SCC from individuals recruited at the Princess Alexandra hospital in Brisbane, Australia, under approved protocols HREC/16/QPAH/364 and HREC/11/QPAH/477. Sterile swabs were first wetted with sodium chloride solution and then firmly rotated over the sampling area for 30 s. Then swab heads were collected in sterile glycerol–saline solution and stored immediately at −80 °C. Suspected IEC and SCC lesions were excised after sampling and the diagnosis confirmed via histopathology.

### 2.2. Isolation of Staphylococcus Clinical Strains

The glycerol-preserved swab samples were plated onto *Staphylococcus* genus-selective mannitol salt agar (MSA) and left to grow for 48 h at 36 °C. Golden colonies with yellow halo on the red MSA agar are indicative of the *S. aureus* species, and thus were picked and restreaked. The next day, single colonies were transferred into tryptic soy broth and grown with shaking at 36 °C overnight. The following day, part of the live bacterial suspension was stored in 100% glycerol (1:1). For DNA extraction and sequencing, the remaining bacteria in suspension was pelleted by centrifugation and resuspended in lysis buffer: 500 mM NaCl, 50 mM TRIS-HCl pH 8.0, 50 mM EDTA, 4% sodium dodecyl sulfate. The glycerol stocks and bacteria cells resuspended within lysis buffer were stored at −80 °C.

### 2.3. Species Identification

To identify species, Gram stains, and coagulase and catalase tests were performed on MSA-positive isolates using standard protocols (Moyes, Reynolds and Breakwell, 2009; Reiner, 2010). Isolates with positive result in all three tests were assayed for the *S. aureus*-specific *nuc* gene via PCR using primers as previously published [10,14]. DNA was collected from liquid cultures of the chosen isolates via thermal extraction method: Pelleted bacteria from overnight cultures were resuspended in 200 μL of ultrapure water, and then incubated at 100 °C. After 10 min of heating, the lysates were cooled on ice and then centrifuged at 10.000× *g* for 3 min. 2 μL of the supernatant were used in a 15 μL PCR assay (0.1 μL Taq DNA polymerase, 20 μM of *nuc* forward 5′-GCGATTGATGGTGATACGGTT-3′ and *nuc* reverse 5′-AGCCAAGCCTTGACGAACTAAAGC-3′ in standard Taq buffer) with the following PCR cycle settings: 94 °C for 2 min, followed by 37 cycles of 94 °C for 1 min, 55 °C for 30 s and 72 °C for 1.5 min, and lastly 72 °C for 3.5 min followed by a 4 °C hold. The 263-bp PCR product was visualized on a 1.5% agarose gel. Finally, the *nuc*-positive isolates were confirmed as *S. aureus* via 16S rRNA gene sequencing. To purify DNA for sequencing, bacteria stored frozen in lysis buffer were thawed and DNA extracted using the Maxwell^®^ 16 MDx instrument (Promega). The 16S rRNA gene was amplified using 27F 5’-AGAGTTTGATCCTGGCTCAG-3’ and 1492R 5’-GGTTACCTTGTTACGACTT-3’ primers. The reaction mixture was purified and cleaned with the Agencourt^®^ CleanSEQ^®^ kit according to standard protocol. Sanger sequencing was performed on the PCR product and the amplicon sequence used to query the GenBank database. Isolates that were catalase, coagulase and *nuc* PCR-positive, Gram-positive cocci and were confirmed *S. aureus* via 16S RNA gene sequencing, were included in this study and selected for secretome production. Although our primary aim was to identify and bank *S. aureus* strains, we also proceeded testing other isolates to use as skin lesion-derived commensal controls. Six isolates that were identified as *S. capitis* and *S. epidermidis,* alongside all collected *S. aureus* isolates, were selected for secretome production and testing.

### 2.4. Collection of Bacterial Secretome Samples

Clinical isolates in glycerol stock were plated onto agar and grown for 24 h at 37 °C. A single colony was then inoculated in chemically defined staphylococcal medium (DSM; recipe in Krueger et al., 2022 [13]) and grown with shaking at 200 rpm at 37 °C. The next day, the optical density at 600 nm (OD600) was measured, and a calculated volume of the overnight culture was used to inoculate 100 mL DSM to provide an initial OD600 of 0.15. These cultures incubated at 37 °C for 24 h on a shaking platform and growth monitored regularity by OD600 measurements. After the final OD600 reading at 24 h, the culture was clarified by centrifugation (3000× *g*; 20 min) and the supernatant filter-sterilized through 0.2 μm regenerated cellulose (Corning; CLS431222) and partitioned into single-use aliquots and stored immediately at −80 °C. Uninoculated DSM was processed the same way to use as a negative control. The sterility of the secretome samples was confirmed by plating aliquots onto agar and monitoring for growth. For experiments, aliquots were freshly thawed, used immediately and discarded after use.

### 2.5. Characterization of S. aureus Isolates

Genome sequencing and multilocus sequence typing of *S. aureus* isolates was performed as described in our previous publication [13]. A phylogenetic tree was constructed for the *S. aureus* strains, based on a core alignment of single nucleotide polymorphisms (SNPs) followed by the removal of recombinant regions. Strain genomes were first aligned against the *S. aureus* USA300 reference genome (Genbank accession GCA_000013465.1) using Parsnp (ver. 1.5.6). Predicted SNPs for each strain were then integrated into the reference genome to generate strain-specific pseudogenomes. Recombinant regions were identified and removed from the alignment of the pseudogenomes using Gubbins (ver. 3.0.0). A maximum-likelihood phylogenetic tree was constructed using RAxML (ver. 8.2.12) using a general time-reversible nucleotide substitution model with gamma correction for site variation (GTRGAMMA) and 1000 bootstraps. The phylogenetic tree was visualized using ggtree (ver. 3.0.4) in R (ver. 4.1.3).

To determine protein expression profiles of *S. aureus*, we performed shotgun mass spectrometry on the *S. aureus* secretome samples, as described in [13].

### 2.6. Culture of Primary Human Keratinocytes

Primary human keratinocytes were isolated from abdominal skin and foreskin collected at the Princess Alexandra Hospital Dermatology Centre (Brisbane, Australia) with patient consent and institutional approval (HREC/11/QPAH/477). Primary keratinocytes were cultured in serum-free DermaLife K keratinocyte basal medium supplemented with 10 µM Rho Kinase inhibitor Y-27632 (Sigma Aldrich, Castle Hill, NSW, Australia; SCM075). Experiments on primary keratinocytes were performed up to passage eight. To explore the effects of *S. aureus* secreted compounds on human keratinocytes, the keratinocytes were cultured in *S. aureus* secretome, or DSM as control, diluted in antibiotic-free DermaLife medium. The MTT assay was used to establish the dose of *S. aureus* secretome that did not compromise cell viability and growth.

### 2.7. MTT (3-(4,5-Dimethylthiazol-2-yl)-2,5-diphenyltetrazolium Bromide) Assay

The MTT assay as developed by Mossmann (1983) was employed to assess keratinocyte cell activity. In brief, 1 × 10 ^4^ keratinocytes were seeded per well of a 96-well plate and left overnight to allow cells to adhere. Keratinocytes were then exposed to *S. aureus* secretome diluted in keratinocyte medium. After 24 h, culture media was replaced with phenol-free media containing 0.5 mg/mL MTT solution (Invitrogen™; M6494). After 4 h incubation at 37 °C, the purple formazan crystals were dissolved with 60 μL dimethyl sulfoxide, and the plates were incubated at 37 °C for a further 10 min. The plates were then shaken for one minute on a plate shaker, and the absorbance was measured at 540 nm. Untreated keratinocytes and keratinocytes killed with lysis solution (Promega, G1821) were used as reference (untreated cells = 100% activity; lysed cells = 0% activity).

### 2.8. Keratinocyte Transcriptomics and Proteomics Experiments

Six biological replicates (3 × foreskin, 3 × abdominal; each cell line from a different donor) in two technical replicates were seeded in T25 flasks. *S. aureus* secretome, or DSM as control, were diluted 1:100 in antibiotic-free DermaLife medium and added immediately after seeding of the cells. The diluted *S. aureus* secretome/DSM was replaced every 24 h for up to 9 days. When cultures reached 70–80% confluence, the cells were dislodged with 0.25% Trypsin-EDTA (Gibco) and reseeded on a new T25 flask (split ratio 1:4). The *S. aureus* secretome or DSM were again immediately added to the freshly passaged cultures. The cultures were harvested once the coincubation with *S. aureus* secretome/DSM had lasted a minimum of 150 h and the culture reached approximately 80% confluence. Depending on the growth rate of the individual cell lines, the total coincubation length stretched over a time span of 150–200 h which correlated to 2–3 cell passages. One technical replicate of the keratinocyte cultures was harvested directly in 600 μL lysis buffer (Buffer RLT, QIAGEN) after two cold PBS washes, immediately frozen on dry ice and stored at −80 °C until further processing for RNA sequencing. The second technical replicate of each keratinocyte culture was washed with warm PBS, dislodged with trypsin, and stored frozen (−80 °C) as a cell pellet allowing for later sample processing for shotgun proteomics.

### 2.9. RNA Sequencing and Bioinformatics Analysis

For RNA purification, the QIAGENs RNeasy mini kit was used according to the manufacturer’s protocol. RNA integrity was assessed on the Agilent RNA ScreenTape System. None of the RIN scores were less than 8, with most being 9.7–10.0. For each sample, 200 ng of RNA starting material was used for library preparation. The TruSeq stranded mRNA library prep workflow was followed as publicly available on the Illumina website. Libraries were then denatured and diluted (after pooling all libraries into a 10 nM pool) according to the Standard Normalization Method (protocol A) as described in the Illumina ‘NextSeq System Denature and Dilute Libraries Guide’ available online. Samples were sequenced on the Illumina Nextseq 550 system at the QIMR Berghofer sequencing facility.

Adapters were trimmed and flanking “N” bases removed from paired-end reads using cutadapt (ver. 1.9). Quality assessment of trimmed files was performed with FastQC (ver. 0.11.8) [15] and then aligned to the ICGC human reference genome (GRCh38, v98) using STAR (ver. 2.7.1a). Read groups for each library were merged using samtools (ver. 1.9). Expected read counts were calculated using featureCounts from Subread (ver. 1.6). Expected counts were matched to gene/transcript annotation information using EnsDb.Hsapiens.v86 annotation package in Bioconductor. A non-linear strategy, the trimmed mean of M values (TMM) method, was used for normalization [16]. All counts were transformed to logCPM (count per million). Only those genes that had at least three CPM across a minimum of three samples (# of replicates) were kept. Differential gene expression was calculated with the R package edgeR using the GLM approach. An adjusted *p* < 0.01 was the cutoff for significance. Pathway enrichment was performed using the fgsea package. Briefly, an enrichment score was calculated for each pathway, and a *p*-value was estimated by permutation analysis. *p*-values were adjusted using Benjamini–Hochberg to correct for multiple hypotheses testing and an adjusted *p* < 0.05 was considered significant.

### 2.10. Shotgun Proteomics on Keratinocyte Cell Lysates

Cells were lysed in freshly prepared lysis buffer (100 mM Tris pH 8.5, 1% sodium deoxycholate, 40 mM 2-chloroacetamide, 10 mM Tris (2-carboxyethyl) phosphine) and sonicated. The protein concentration of each lysate was quantified by standard bicinchoninic acid assay. The 20 samples contained between 4.5 and 9.8 μg protein per μL. Each sample was diluted to 1 μg/μL in lysis buffer and heated to 92 °C for 5 min. Then, 10 μg of each sample was diluted to a final volume of 100 μL with milliQ water and digested overnight with 200 ng of trypsin (Promega; V5111). Reactions were acidified to a final concentration of 0.5% TFA (*v*/*v*), and insolubles removed by centrifugation at 15,000× *g*, 4 °C for 20 min. Peptides in the soluble fraction were desalted using C-18 STAGE tips according to the manufacturer’s instructions (Glygen catalogue Velo-C18-20µg). C-18 eluents were vacuum dried and resuspended in 20 µL of 0.5% TFA.

Samples were resolved on a Thermo U3000 nano-HPLC system and analysed on a Thermo Q Exactive Plus Orbitrap mass spectrometer. The HPLC setup used a C-18 trap column (DX160454) and a 50 cm EasySpray^TM^ C-18 analytical column (ES803A) from Thermo Fisher Scientific^TM^. Mobile phases were A: 0.1% formic acid, and B: 80% acetonitrile with 0.1% formic acid. 2 µL of sample equivalent to 1 μg of peptides were loaded in 2% B, and peptides eluted over a gradient from 2–30% B over 64 min, followed by 30–50% B over 10 min at 250 nL/min. An EasySpray source was ran in positive ion DDA mode with settings typical of peptide analyses. Briefly, full MS scans were acquired at 70,000 resolution, automatic gain control target was 3 × 10 ^6^, with a maximum injection time of 100 ms. MS2 fragmentation was carried out on the top ten precursors, excluding 1 + precursors. Precursor isolation width was 1.4 m/z and normalized collision energy was 27. MS2 resolution was 17,500 with an automatic gain control target of 5 × 10 ^5^. Maximum injection time was 50 ms and the exclusion window was 10 s.

Raw data files were analysed with MaxQuant (ver. 1.5.8.3) [17] which assembled proteins by matching peptides to the human reviewed UniProtKB proteome database (April 2020; 20,365 proteins). From the resulting list of 4114 identified proteins, potential contaminants, proteins with a score < 5, and proteins with only one or without unique peptides, were removed. Quantile normalization and missing value imputation was performed on the remaining filtered proteins (*n* = 3153). Proteins which were missing in < 25% of all samples were considered missing at random and imputed using localized least square regression (llsimpute) as described elsewhere [18]. Proteins missing in > 25% were imputed with the minimum detected value (values drawn randomly from a normal distribution centred at sample minimum and with SD estimated from nonmissing proteins). Log2 transformed data was analysed using the R limma package to identify statistically significant proteins (adjusted *p* value < 0.05). The fgsea package in R was used for pathway enrichment analysis using logFC as a ranking statistic and pathways from the Reactome database [19].

### 2.11. Measurement of Intracellular ROS

ROS were quantified using ROS-sensitive fluorescent probes 2′,7′-dichlorofluorescin diacetate (DCF-DA), totalROX [20] and 4,5-diaminofluorescein diacetate (DAF-2 DA). TotalROX was kindly provided by Haolu Wang (University of Queensland Diamantina Institute). The DCF-DA and DAF-2 DA were purchased from Sigma-Aldrich (D6883) and abcam (ab145283), respectively. The probes were dissolved in DMSO to a 10 mM working stock, which was divided into small aliquots and stored at −20 °C. Keratinocytes were seeded in 96-well tissue culture-treated black plates in DermaLife medium and allowed to adhere overnight. Cells were then washed twice with warm PBS and 100 μL of either 10 μM DCF-DA, DAF-2 DA or TotalROX in PBS was added. To allow keratinocytes to internalize the probe, the plate was incubated in the dark for 30 min (except DAF-2 DA for 60 min) at 37 °C. To remove residual probe, cells were washed three times with warm PBS. Then the controls AAPH (4 mM) and H_2_O_2_ (100 μM) and *S. aureus* secretomes +/− NAC (2 mM) were added (all diluted in PBS) and the fluorescence signal measured over time on a CLARIOstar microplate reader (BMG Labtech). DCF-DA is converted to DCF by ROS which was measured at λexcitation and λemission (λex/λem) of 485 nm and 530 nm, respectively. DAF-2 DA is deacetylated by intracellular esterases to DAF-2 which reacts with nitric oxide to yield fluorescent triazolofluorescein (DAF-2T) detected at λex/λem of 490/515 nm. The oxidized product of totalROX was monitored at λex/λem 640/670 nm.

As a probe-independent method to assess nitric oxide, the Griess assay was performed [21], which measures its stable breakdown product nitrate. Primary human keratinocytes were seeded in 96-well plates and allowed to adhere overnight. The cells were then challenged with *S. aureus* secretome diluted in serum- and antibiotic-free DermaLife medium. After 24 h, 70 μL of culture supernatant was transferred into a clean microplate and equal volume of Griess reagent (Sigma-Aldrich; G4410) added. After ten minutes of incubation at room temperature in the dark, absorbance was read at 540 nm.

### 2.12. Assessment of Histone H2A.X Phosphorylation via Immunofluorescence Staining

Normal human keratinocytes from adult abdominal skin from five different donors were seeded in 24-well plates containing cover slips at 0.05 × 10 ^6^ in technical duplicates. After overnight incubation, keratinocytes were treated with 10 µM camptothecin, a positive control known to induce DNA damage, or secretome from *S. aureus*, *S. epidermidis*, or *S. capitis* clinical strains isolated from either IEC or SCC, diluted 1 in 50 in antibiotic-free Dermalife medium. Untreated cells and cells exposed to uninoculated bacterial media DSM served as negative controls. After 6 h, the cells were washed with PBS and then fixed in 4% paraformaldehyde for 20 min. Cells were washed again, followed by an incubation in 2% BSA-PBS with 0.5% Triton X100 for 60 min at 37 °C. Following permeabilization and blocking, the primary antibody, anti-phospho-histone H2A.X (Ser139, Sigma 05-636), was added. After 60-min incubation at 37 °C, cells were washed with PBS and the secondary antibody (AlexaFluor488) was added. After 60 min, the cells were washed again, and stained with DAPI for 5 min at room temperature. The cover slips were then mounted, and γH2A.X foci were visualized and images acquired on a Zeiss 780 NLO confocal microscope with a 63X objective. The open-source software QuPath 0.2.3 was used for automatic cell detection and γH2A.X foci quantification.

### 2.13. 8-Hydroxy-Deoxyguanosine (8-OHdG) ELISA

Normal human keratinocytes were cultured in DermaLife medium and 8-OHdG experiments were carried out in T75 flasks on cultures between 70–90% confluence. Cells were exposed to *S. aureus* secretome (1 in 100; 1 in 200) and positive controls CuO (1 μg/mL) and H_2_O_2_ (200 μM) diluted in antibiotic-free Dermalife medium for six and 200 h. For the 200-h experiment, the medium was replaced with fresh stimulant every 24 h, and cells were split regularly to avoid confluence. At end point, cells were washed with PBS, dislodged with trypsin, and harvested as a cell pellet which was snap frozen on dry ice and stored at −20 °C until further processing. The following procedures were carried out rapidly in small sample sets to avoid extended periods of exposure to oxidizing agents. To extract DNA, cells were thawed and processed according to the protocol provided for the DNeasy Blood & Tissue Kit (QIAGEN, Cat No. 69506) including an RNA digestion step with RNase A (QIAGEN, Cat No. 19101). DNA was eluted in 50 μL ultrapure molecular grade water. DNA concentration was determined in duplicate initially by NanoDrop™ spectrophotometer using A260/A280 ratios and confirmed via the broad range Invitrogen™ Quant-iT™ dsDNA Assay Kit (Cat No. Q33130). The DNA amounts within sample sets were standardized by diluting DNA samples in ultrapure water to the desired concentration (2.5 or 3.5 μg). The DNA was then digested using the New England BioLabs Nucleoside digestion mix (Cat No. M0649S). Each reaction used 1 μL of enzyme, and enzymatic digestion was allowed to proceed for 4 h at 37 °C on a shaking platform (300 rpm). For DNA damage assessment, an 8-OHdG ELISA was carried out immediately after digestion (Abcam Ab201734). A standardized amount of DNA digest (2.5 or 3.5 μg) were diluted in the provided sample diluent buffer to a 50 μL final volume. The remaining assay procedure was followed according to the manufactures’ protocol.

### 2.14. Quantification of Phenol-Soluble Modulin (PSM) Toxins

Selected custom-synthesized (Mimotopes, Clayton, Australia) phenol-soluble modulins (PSMs) from *S. aureus* (deltatoxin: fMAQDIISTIGDLVKWIIDTVNKFTKK, PSMalpha1: fMGIIAGIIKVIKSLIEQFTGK) were used to experimentally determine fragmentation behaviour which was then employed to predict transitions in silico for PSM peptide sequences of other *Staphylococcus* species obtained from the literature [22,23]. PSM peptides were most likely to produce multiple charged precursor ion species (e.g., charge state 3+, 4+) in the mass range of *m/z* 600–900 with considerable signal intensities. PSMs tend to form y1 to y5-fragment ions above the precursor mass with one charge less than the precursor. PSMs also showed singly charged b2 to b6-ions upon fragmentation. In addition, a list of truncated PSM peptides (dPSM) was added to the transition list according to observations described previously [24,25,26]. A comprehensive transition list (Table S3) containing 73 full length and truncated peptides and 622 transitions from *S. aureus, S. epidermidis and S. capitis* was compiled. Prior to running samples, this list was assessed for the presence/absence of peptide species in current sample matrices by running strain-specific sample pools with strain-specific transition lists. Following the analysis, the final MRM assay contained 132 transitions from 32 peptide species (top 3–5 transitions per peptide; Table S4) and with a dwell time of 5 ms resulted in a cycle time of 980 ms.

Prior to LCMS analysis, culture supernatant samples were diluted to 60% 2,2,2-trifluoroethanol (TFE) and 1% trifluoroacetic acid (TFA). Following centrifugation, a supernatant aliquot of 5 µL was loaded onto the column. Note, a high concentration of strong solvent like TFE was required to keep PSMs in solution and achieve good intra- and interday injection reproducibility during autosampler storage.

PSM peptides were analysed on a 1290 Infinity II UHPLC coupled to a 6470 QQQ mass spectrometer via AJS ESI source (Agilent, Santa Clara, CA, USA). The mass spectrometer was operated in positive ionization mode acquiring data in multiple reaction monitoring (MRM). Quadrupoles 1 and 3 were set at unit resolution. Source conditions were as follows: Gas temperature 270 °C, gas flow 11 L/min, sheath gas temperature and flow at 400 °C and 12 L/min, respectively, nebulizer 35 psi, fragmentor 175, capillary voltage at +4500 V, nozzle voltage was 500 V. Collision energy (CE) was calculated using the default linear equation for all charge states: CE = 0.036 × (*m*/*z*) − 4.8. Previously optimized CE values for synthesized peptides showed a good agreement with predicted values from the equation.

Peptide separation was achieved on a Poroshell 120 EC-C18 (2.7 µm, 2.1 × 50 mm; Agilent) analytical column connected to a 2.1 × 5 mm guard column of the same resin. Note, longer columns and fully porous column material caused significant PSM loss on the column. The autosampler and column temperature were set to 4 °C and 50 °C, respectively. Mobile phases A and B were milliQ water and acetonitrile, both containing 0.1% formic acid, respectively. Total method runtime was 10 min with the following gradient employed: 0 min (1% B)-3 min (1% B)-7.5 min (100% B)-8 min (100% B)-8.1 min (1% B)-10 min (1% B). The flow was diverted to waste for the first three minutes and a flow rate of 0.5 mL/min was applied. All samples were run in technical triplicates in a randomized order.

MRM data was analysed in Skyline software [27]. All peaks were manually checked for correct peak picking and integration. Transition results were exported (Table S5), further analysis and graph plotting was performed in GraphPad Prism.

## 3. Results

### 3.1. RNA-Seq and Proteomics on Human Keratinocytes after Challenge with S. aureus Secretomes

**Figure 1:**
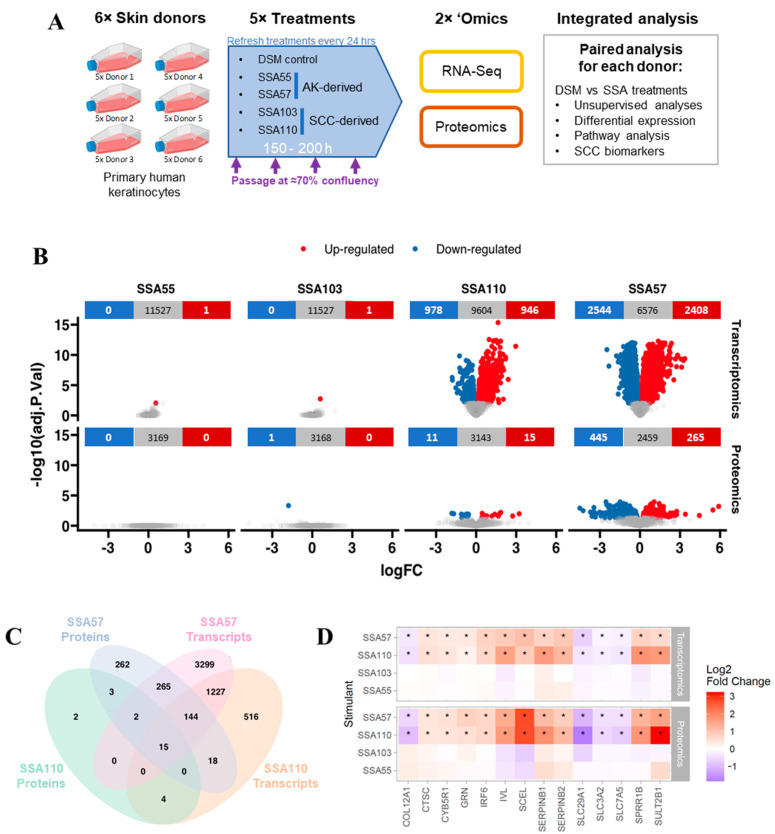
Heterogenous impact of *Staphylococcus aureus* secretomes on primary human skin keratinocyte transcriptome and proteome. (**A**) Experimental workflow schematic. Primary keratinocytes from six different donors were cultured and treated with secretome from four different *S. aureus* isolates (designated SSA55, SSA57, SSA103, SSA110), sampled from actinic keratosis (AK) or squamous carcinoma (SCC) lesions as indicated. The bacterial cell culture medium (defined staphylococcal medium, DSM) was used as a negative control for each donor keratinocyte cell line. Treatments were refreshed every 24 h, and cells were passaged at ~70% confluency. After up to 200 h of stimulation, keratinocytes were harvested for RNA sequencing and proteome profiling. (**B**) Volcano plots showing the distribution of log2 fold changes (logFC) and significance (adjusted *p*-value) in gene expression (top) and protein expression (bottom) in keratinocytes treated with *S. aureus* secretomes compared to the DSM control (mean of six biological replicates). Using a cutoff of *p* < 0.01, significantly up- or downregulated genes/proteins are coloured in red or blue, respectively. (**C**) Venn diagram showing the total number of differentially expressed genes/proteins for SSA57 and SSA110 and overlap between conditions. (**D**) Heatmap showing the fold change of the 14 differential genes/proteins consistent across transcriptome and proteome for both SSA57 and SSA110, with data from SSA53 and SSA103 shown for comparison. * *p* < 0.05.

To gain insights into whether *S. aureus* overabundance on photodamaged skin might promote skin cancer development and progression, we first employed a nontargeted approach to broadly explore the effects of *S. aureus* secreted compounds on cellular processes and cancer pathways in cultured human skin cells. Primary keratinocytes derived from six skin donors were exposed to lesion-derived *S. aureus* secretomes as well as uninoculated defined staphylococcus medium (DSM; negative control) and monitored for changes in their transcriptome and protein expression (Figure 1A). To account for potential donor-specific responses, fold change from DSM control was calculated for each secretome for each keratinocyte cell line prior to differential expression and pathway enrichment analyses.

Since we, and others [13,28] have shown that there is a large heterogeneity across *S. aureus* strains, we selected four genetically and phenotypically distinct strains from 34 lesion-associated clinical isolates characterized by multilocus sequence typing, phylogenetic analysis and secretome protein expression profiles to test in the RNA-Seq and proteomics experiments: isolates SSA55 and SSA57 from AK skin, and SCC-derived SSA103 and SSA110 (Figure S1). This analysis also showed no particular strains to be more commonly found on skin cancers.

### 3.2. Gene and Protein Expression in Keratinocytes Is Strongly Altered by Some S. aureus Secretomes

Differential expression analysis showed that the secretome of isolates SSA57 and SSA110 induced substantial changes to the keratinocyte transcriptome and proteome, while SSA55 and SSA103 treatment had little impact on gene and cellular protein expression (Figure 1B, Table S1). SSA57 produced the largest effect with 4952 genes and 710 proteins significantly altered, while SSA110 produced significant changes in the expression of 1924 genes and 26 proteins (Figure 1B). We evaluated the overlap between the differentially regulated transcriptome and proteome, noting the caveat of the lower coverage of the cellular proteome compared to the transcriptome, due to the broader dynamic range of the proteome, and the fact that secreted proteins were not measured. While not all transcriptome changes are expected to translate to proteomic changes, we found that 426 and 26 differentially expressed proteins had differentially expressed transcripts of the same direction of change for SSA57 and SSA110, respectively (Figure 1C). Furthermore, 15 genes and their corresponding proteins were significantly altered by both SSA57 and SSA110, 14 of which showed unidirectional change (Figure 1C), including downregulation of nucleoside and amino acid transporters (SLC29A1, SLC3A2, SLC7A5), and upregulation of markers associated with keratinocyte distress and inflammation (SERPINB1, SERPINB2, GRN, CTSC) and premature skin ageing (IVL, SCEL, SERPINB2; SULT2B1) (Figure 1D). The upregulation of SCC biomarker *ctsc* encoding Cathepsin C, a nonredundant mediator of squamous carcinogenesis involved in angiogenesis and immune regulation in neoplastic skin [29] was of particular note.

### 3.3. S. aureus Mediates Upregulation of Several SCC Biomarkers in Primary Human Keratinocytes

**Figure 2:**
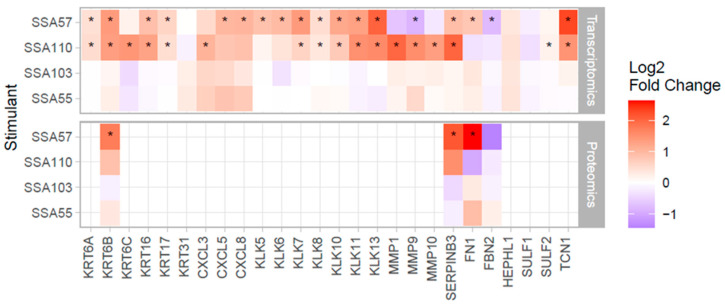
*S. aureus* secretomes increased expression of SCC biomarkers in human keratinocytes. Heatmaps show the gene (top) and protein (bottom) expression changes of biomarkers linked with SCC progression [30,31,32,33,34,35] in keratinocytes challenged with *S. aureus* secretome compared to control. * *p* < 0.05. Note, most of the biomarkers are highly secreted proteins, hence, they were not detected by shotgun proteomics of the cellular fraction.

We next examined the effects of the *S. aureus* secretomes on keratinocyte expression of other well-known SCC biomarkers [30,31,32,33,34,35]. The secretomes of both SSA57 and SSA110 significantly increased transcription of genes encoding kallikrein serine proteases and keratins, factors that promote inflammation and keratinocyte proliferation, migration and invasion [30,33,36] (Figure 2). Further, *S. aureus* secretomes induced gene expression of multiple CXC chemokines involved in SCC progression. SSA110 secretome significantly increased mRNA levels of matrix metalloproteinases that enhance angiogenesis and promote tumour invasion and metastasis [31,32], and SSA57 secretome upregulated mRNA and protein expression of SCC biomarkers serpin B3 and fibronectin 1. With the exception of KRT6B, SERPINB3, FN1, FBN2, these SCC biomarkers were not detected at the protein level, likely because most are secreted proteins with low abundance in the cell lysate. Nevertheless, the four detected proteins validated the direction of change of the transcriptome data, albeit not all reaching significance.

### 3.4. S. aureus Secretome Downregulates Cell Cycle and DNA Repair and Induces Oxidative Stress Markers in Primary Human Keratinocytes

**Figure 3:**
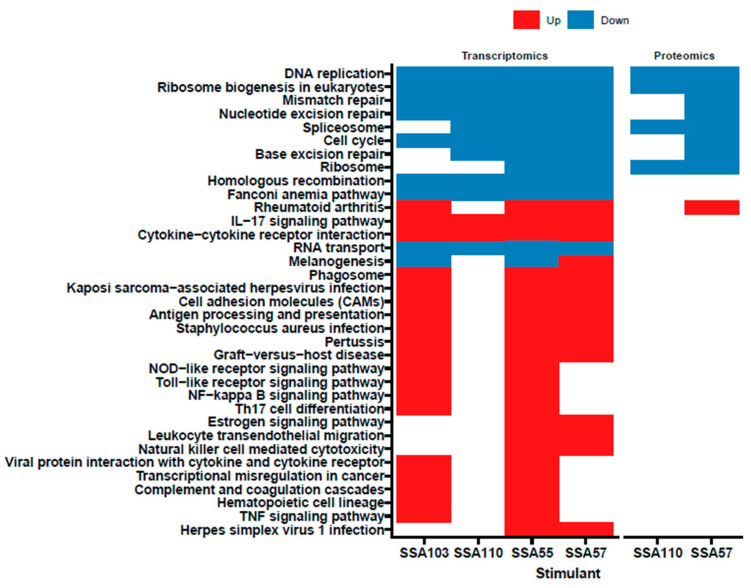
Pathway enrichment of transcripts and proteins informs cellular changes in response to challenge with *S. aureus* secretomes. Enrichment of biological pathways in keratinocytes based on the changes in genes (left panel) and proteins (right panel) was evaluated using the KEGG database. All differentially expressed pathways of significance (i.e., adj. *p* < 0.05) are shown and the direction of differential expression is indicated by colour of the box (blue = downregulated; red = upregulated). No significant pathways were found for SSA55 and SSA103 based on the proteomics data. Detailed pathway analysis results based on the Reactome database are available in Table S3.

Pathway enrichment analyses were conducted to evaluate functional changes in keratinocytes induced by the *S. aureus* secretomes (based on KEGG database Figure 3 and Reactome database Table S2). At the transcriptome level, numerous proinflammatory pathways were activated in keratinocytes by *S. aureus* secretomes, particularly by secretomes from SSA55 and SSA103 (Figure 3). In addition, the transcription of several pathways was significantly downregulated by all four *S. aureus* secretomes tested, including DNA replication, RNA transport, ribosome biogenesis and cell cycle (Figure 3, Table S2). A number of these keratinocyte pathway changes were validated at the protein level for SSA57 and SSA110 treatment, however, no significant changes in protein pathways were observed with SSA55 or SSA103. The repression of genes involved in the cell cycle is commonly initiated in response to cell stressors such as oxidative stress and DNA damage [37]. In line with this, several pathways indicating the presence of oxidative stress and DNA damage in keratinocytes were significantly upregulated by the SSA57 and SSA110 secretomes (Table S2). Oxidative stress-mediated DNA damage can be mitigated by endogenous DNA repair [38]. Interestingly, the pathway analyses indicated the downregulation of numerous DNA repair mechanisms, including homologous recombination, mismatch, base excision, nucleotide excision and double-strand break repair (Figure 3, Table S2). Additionally, the Fanconi anaemia pathway, crucial for the efficient repair of damaged DNA and to preserve genomic integrity [39], was significantly downregulated by all four tested *S. aureus* secretomes (Figure 3). Based on these observations, we hypothesized that exposure of keratinocytes to *S. aureus* secretomes might lead to oxidative stress and reactive oxygen species (ROS)-dependent DNA damage that keratinocytes are incapable of repairing. Thus, we next sought to validate the suspected ROS-inducing and genotoxic ability of *S. aureus*, testing a larger number of clinical isolates in additional keratinocyte in vitro experiments.

### 3.5. S. aureus Secreted Factors Trigger Oxidative Stress in Primary Human Keratinocytes

**Figure 4:**
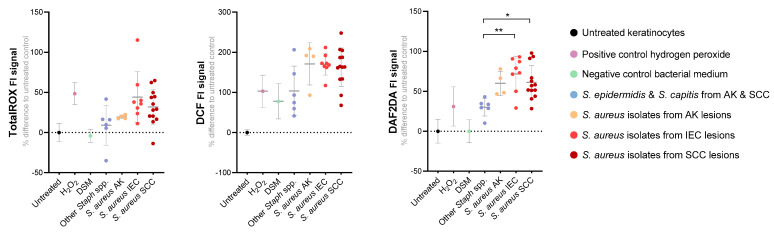
Exposure to *S. aureus* secretome increases intracellular reactive oxygen and nitrosative species in human keratinocytes. The level of intracellular reactive oxygen/nitrogen species after 6 h treatment with secretome from *S. aureus* isolated from actinic keratosis (AK, n = 4), intraepithelial carcinoma (IEC, n = 8) or squamous cell carcinoma (SCC, n = 13), was measured in primary human keratinocytes using three different fluorescent probes. The positive control was hydrogen peroxide (H_2_O_2_) and negative controls included untreated cells, *S. aureus* culture media (DSM) and secretome from other *Staphylococcus* species derived from AK and SCC (*S. epidermidis*, *S. capitis*). Displayed is the relative fluorescent signal (as percentage difference from the untreated control) for the TotalROX probe measuring peroxyacids, free radicals, singlet oxygen and nitrosative species, DCF-DA probe measuring hydroxyl and peroxyl radicals and DAF-2 DA probe, a nitric oxide indicator. Each dot represents a different bacterial isolate and shows the grouped mean value from three independent experiments, each performed in technical triplicates. Dunn’s multiple comparisons test was performed between groups *S. aureus* AK, IEC, SCC and other *Staphylococcus* spp. for each assay (* *p* < 0.05; ** *p* < 0.01).

Changes in keratinocyte intracellular ROS levels after exposure to *S. aureus* secretomes were assessed using three ROS-sensitive fluorescent probes. The TotalROX system was used to measure per-acids, radicals, singlet oxygen, and nitrosative species [20], DAF-2 DA probe was used to measure nitric oxide, and DCF-DA probe for hydroxyl and peroxyl radicals and other ROS. Probe-loaded primary human keratinocyte cultures were challenged with secretomes from 25 *S. aureus* isolates from AK, IEC, or SCC lesions of 15 subjects. We found certain *S. aureus* secretomes were more potent ROS inducers than others, as intracellular ROS signals increased between 20–250% in keratinocytes following exposure (Figure 4). A time- and dose-dependent effect was observed, and the positive signal was confirmed to be solely ROS-mediated as the addition of a ROS-neutralizing antioxidant caused the loss of signal (Figure S2A,B). Keratinocyte ROS levels were generally higher in response to secretomes from lesion-associated *S. aureus*, as compared to those from other *Staphylococcus* species isolated from AK and SCC (Figure 4). As independent validation, we confirmed the DAF-2 DA probe/nitric oxide results by quantifying a stable nitric oxide breakdown product using the Griess assay (Figure S2C). The measured intracellular ROS in the keratinocytes could either originate from the keratinocytes themselves, and/or external ROS present in the secretome as a byproduct of culturing *S. aureus*. To determine the source of ROS, we also assayed for ROS in the bacterial secretomes using the same three probes. No or minimal positive signal was observed in *Staphylococcus* secretomes incubated with DAF-2 DA, suggesting that nitric oxide was produced primarily by keratinocytes. Stronger ROS signals were observed with TotalROX and DCF probes indicating the presence of some ROS in *Staphylococcus* secretomes. However, *S. aureus* secretomes retained a strongly ROS-promoting effect on keratinocytes even after the ROS naturally present in secretomes were dissipated, confirming that that more stable compounds secreted by *S. aureus* can elicit keratinocytes to generate high levels of DCF-detectable ROS (Figure S3).

### 3.6. S. aureus Products Compromise the Integrity of DNA in Human Keratinocytes

**Figure 5:**
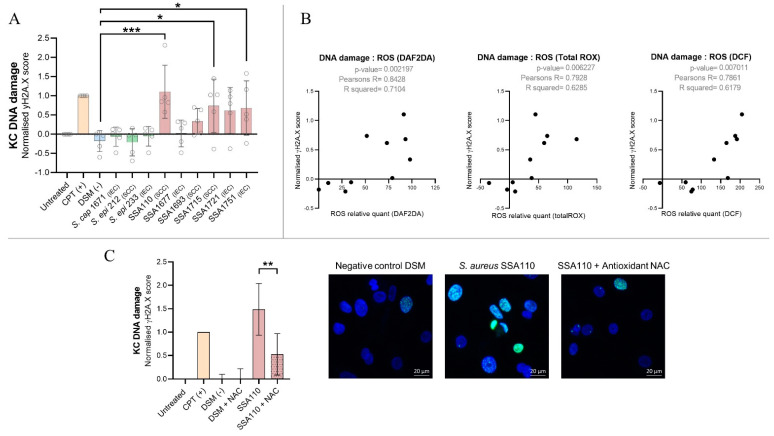
*S. aureus* causes keratinocyte DNA double strand breaks, and *S. aureus* secretome-induced ROS levels positively correlate with genotoxicity in human keratinocytes. (**A**) Primary human keratinocytes from five different donors were grown on coverslips in technical duplicates and exposed to the secretomes prepared from *Staphylococcus* species isolated from intraepithelial carcinoma (IEC) or squamous cancer (SCC) skin for 6 h. Negative controls included no treatment and Staphylococcus culture media (DSM). Positive control was the DNA breakage promoting drug camptothecin (CPT, 10 µM). Histone H2A.X phosphorylation (γH2A.X, green) was measured via immunofluorescence staining as a measure of DNA double strand breaks. DAPI (blue) was used to stain the nuclei. Images were captured at 63× magnification by confocal microscopy (representative images are shown in panel C. The number of foci per cell was enumerated and calculated to a score normalized to the control (0) and positive control CPT (1). Statistical significance was determined by Dunnett’s multiple comparisons test (* *p* < 0.05; *** *p* < 0.001). (**B**) Pearson’s correlation analysis was conducted to examine the correlation between keratinocyte intracellular ROS and DNA damage induced by Staphylococcal secretomes. (**C**) To determine if *S. aureus*-induced DNA damage in keratinocytes is ROS-mediated, primary human keratinocytes from two different donors were exposed to the genotoxic *S. aureus* secretome from isolate SSA110 +/− antioxidant N-Acetyl Cysteine (NAC, 2 mM). Statistical significance between SSA110 and SSA110 + NAC treated cells was determined by paired *t*-test (** *p* = 0.0025).

We demonstrated that *S. aureus* products increase keratinocyte ROS levels and suppress the DNA damage repair response. Therefore, it is plausible that keratinocytes exposed to *S. aureus* undergo oxidative stress and accumulate increased DNA damage and, therefore, are more susceptible to oncogenic mutations and transformation. To investigate this possibility directly, we quantified phosphorylation of histone H2A.X, a marker for DNA double strand breaks, in primary human keratinocytes after six hours exposure to secretome from IEC and SCC-associated *S. aureus*. Genotoxic effects were observed for five of six *S. aureus* secretomes examined, whereas secretomes from IEC and SCC-derived *S. epidermidis* and *S. capitis* did not cause DNA damage (Figure 5A). The most genotoxic *S. aureus* isolate SSA110 increased the frequency of DNA double strand breaks to a level similar to the potent mutagen camptothecin (Figure 5A). The secretome-induced DNA damage was proportionally associated with a loss in cell viability (*p*-value ≤ 0.0001; Pearson’s R = −0.93), however, even after 24 h of treatment with strongly genotoxic *S. aureus* secretomes, the maximum reduction in keratinocyte viability was 36% (Figure S4).

The genotoxic effects of SSA110 were independently validated by measuring the level of 8-hydroxy-2’-deoxyguanosine (8-OHdG), a common product of oxidative DNA damage. Increased levels of 8-OHdG were detected after both six- and 200 h treatment (Figure S5). Keratinocytes continued to proliferate in the presence of SSA110, as seen in the initial 200 h exposure experiment (Figure 1). Altogether, this highlights that *S. aureus* can cause ongoing DNA damage in surviving cells that is not sufficiently repaired over time, thus creating an environment susceptible for oncogenesis.

UV radiation can initiate and promote skin cancers by causing DNA damage mediated by ROS. We found a strong correlation between the *S. aureus*-induced intracellular level of ROS and induced genotoxicity in keratinocytes suggesting *S. aureus* also causes oxidative/ROS-mediated damage of the DNA (Figure 5B). Indeed, the presence of the ROS-scavenging antioxidant N-acetyl cysteine can rescue keratinocytes from *S. aureus* secretome-induced DNA damage (Figure 5C). We found no correlation (*p*-value = 0.48; Pearson’s R = 0.27) between secretome-induced DNA damage and the magnitude of proinflammatory responses in keratinocytes as determined in our previous study [13].

*S. aureus*-derived lytic peptide toxins, PSMs α1 to α4, have previously been shown to cause ROS-dependent DNA damage in human cervical and osteoblast cells [40]. To investigate whether PSM toxins may also play a role in the observed *S. aureus* secretome-mediated induction of ROS and DNA damage in human keratinocytes, we quantified PSMα1-4 and PSM δ-toxin in our staphylococcal secretomes via targeted mass spectrometry (Figure S6). Secretomes with higher concentrations of PSMs induced higher levels of ROS and larger genotoxic effects in keratinocytes, and we observed a significant correlation between the concentration of each tested PSM in secretomes and the various measures of ROS and DNA damage in secretome-treated keratinocytes (Table 1). It is worth noting that the secretome from strain SSA1677, with low PSM content (Figure S6) and no genotoxic effect (Figure 5A), was not an artifact of poor growth and/or low overall protein secretion. The bacterial density at the time of secretome harvest and their respective protein concentrations were comparable across all the strains tested, and no correlation with the assessed readouts (e.g., DNA damage) was observed (Figure S7).

## 4. Discussion

The progression from AK to IEC and SCC is typically a long process taking place over years with increasing accumulation of photodamage and mutations. It is plausible that throughout this time, the skin microbiota may influence progression and directly promote skin carcinogenesis. AK, IEC and SCC have been associated with an increased abundance of *S. aureus* [8,9,10,11,12]. To gain insight into possible *S. aureus*-mediated pro-tumorigenic functions in the skin, cancer-related biological processes were investigated in primary human keratinocytes after challenge with secretomes from lesion-associated *S. aureus* isolates. Evidence presented here supports the notion that at least some *S. aureus* strains secrete compounds that, similar to UV exposure, may contribute to SCC development through the expression of SCC biomarkers, impairing DNA repair and skin barrier function, and causing oxidative stress and DNA damage in the skin.

We found that secretomes from AK and SCC-associated *S. aureus* influence the expression of matrix metalloproteinases (MMPs) and kallikrein serine proteases in primary human keratinocytes. MMPs cause the breakdown of the extracellular matrix, reduce barrier function, and have proangiogenic effects [31,32]. MMP1, MMP3 and MMP10 are typically absent in normal skin, but significantly upregulated in cutaneous SCC [30], and it has been proposed that MMP9 plays a crucial role in the initiation of SCC in immunosuppressed patients [41]. *S. aureus* secretomes from strain SSA110 caused keratinocytes to highly express all four of these SCC-associated MMPs. Similar to MMPs, the overexpression of kallikreins compromises skin barrier integrity due to an increase in endogenous protease activity [42] and some *S. aureus* secretomes were found to significantly induce the expression of numerous kallikreins in human keratinocytes. Supporting these findings, it has been previously shown that sterile culture supernatant from *S. aureus* str. Newman induced the transcriptomic expression of kallikreinsin cultured keratinocytes [43]. By activating the expression of MMPs and kallikreins, some *S. aureus* strains may mediate an impairment of skin barrier function. Since the disruption of the skin barrier is known to promote epidermal hyperplasia and SCC development [44], this may present a mechanism by which persistent *S. aureus* colonization causes susceptibility to skin cancer formation.

*S. aureus* secretomes were found to induce keratinocyte transcription and increased protein levels of antioxidant enzymes that are commonly induced in cells undergoing oxidative stress. It was further validated that *S. aureus* secreted products indeed increase intracellular ROS levels in primary human keratinocytes. Keratinocytes may produce ROS in response to *S. aureus* secretome challenge perhaps as a stress response, or to eradicate the pathogen. Indeed, the latter phenomenon has been reported to occur via *Cutibacterium acnes*-dependent keratinocyte production of superoxide anions [45]. Here, we demonstrate that ROS, and in particular NO, are directly produced by keratinocytes in response to secretome exposure, noting that the secretome also contains ROS as a byproduct of *S. aureus* cultivation. The latter observation is consistent with previous reports that *S. aureus* clinical strains are able to generate ROS [46]. A technical limitation of the oxidative stress experiments is nonspecific signal and auto-oxidation associated with fluorescent ROS probes [47,48]. For this reason, multiple independent probes were tested and since each probe confirmed the presence of ROS, the likelihood of false positive results was minimized. Further, a probe-independent method, the Griess assay, also confirmed the NO results. Lastly, other studies have noted that *S. aureus* is able to stimulate ROS production in other cell types [40,49,50]. For instance, *S. aureus* secreted products were shown to mediate the production of ROS in neutrophils via formyl peptide receptor activation [49] and *S. aureus* infection promoted ROS production in osteoblasts due to Toll-like receptor 9 recognition of unmethylated bacterial CpG-DNA [50].

Eukaryotic cells experiencing severe oxidative stress typically arrest their cell cycle temporarily and upregulate DNA repair systems to facilitate the repair of oxidative lesions [51]. Indeed, transcriptomics and proteomics data indicated an initiation of cell cycle arrest in keratinocytes exposed to *S. aureus* secretomes that could be a stress response and/or a result of the action of cyclomodulins, bacterial toxins with the ability to modulate the eukaryotic cell cycle [52,53,54,55]. Surprisingly, keratinocytes also suppressed their DNA repair pathways in response to *S. aureus* secretome exposure. For instance, the Fanconi anaemia (FA) pathway was downregulated and biallelic mutations in any of the 22 FA pathway genes are known to cause impaired DNA repair, chromosomal instability and predisposition to cancer [39]. One of the FA proteins in particular, FANCA, has been found negatively associated with cutaneous SCC [56]. FANCA, together with other FA proteins, were significantly downregulated in keratinocytes by two of the four tested *S. aureus* secretomes. An effective DNA damage response is critical to preventing cancer development, including cutaneous SCC [57]. *S. aureus*-induced downregulation of DNA damage repair mechanisms, alongside the observed increase in oxidative stress, may facilitate the accumulation of mutations, leaving normal keratinocytes more susceptible to aberrant cellular physiology and transformation. We demonstrated that secreted products from multiple SCC-associated *S. aureus* strains can indeed compromise the genomic integrity of keratinocytes by assessing frequency of histone H2A.X phosphorylation, a marker of DNA double strand breaks, and 8-OHdG lesions, a marker for oxidative DNA damage. This finding is consistent with a recent study showing that intracellular infection with live *S. aureus* can cause ROS-mediated DNA damage in human osteosarcoma and cervical cancer cells, which was shown to be mediated by secreted cytolytic toxins PSMα1-4 [40]. We also identified a significant correlation between secretome PSM levels and keratinocyte DNA damage. Additional studies are needed to validate this association and elucidate how specific component(s) of *S. aureus* secretomes are driving the downregulation of DNA repair and cell cycle.

## 5. Conclusions

Collectively, the results presented herein provide evidence and mechanistic insights into how secreted compounds from *S. aureus* can create an environment favourable for the progression of skin cancer. Although it is indisputable that UV exposure is the main driver of skin carcinogenesis, our study highlights the importance of the skin microbiome in AK, IEC and SCC and strengthens the hypothesis that an overabundance of certain *S. aureus* strains on premalignant skin lesions may act as a pro-tumorigenic stimulus by inducing oxidative stress, DNA damage, and impaired DNA repair. As a next step, it would be interesting to assess the frequency of pro-tumorigenic *S. aureus* strains in larger cohorts of patients from different geographical regions and environmental conditions, and from different anatomical sites. Further, since *S. aureus* in planktonic culture may produce different secreted products and/or at different quantities than during skin colonization, future research will need to establish whether *S. aureus* colonization on the skin mediates the observed pro-tumorigenic effects in vivo. Human clinical trials are needed to investigate whether eradicating *S. aureus* has favourable clinical outcomes, which may ultimately change standard practice and treatment of keratinocyte cancer precursors.

## Figures and Tables

**Table 1:** Secreted phenol soluble modulin (PSM) levels correlate positively with the oxidative and genotoxic ability of *S. aureus* secretomes on human keratinocytes. Targeted mass spectrometry was used to measure phenol soluble modulins α1-4 and d-toxin in secretomes from Staphylococcus assayed in Figure 5A. Pearson’s correlation analysis was conducted to examine the correlation between relative PSM level and ROS or DNA damage.

Variable I	Variable II	Pearson’s R	*p*-Value
δ-toxin	γH2A.X	0.94	<0.0001
PSMα1	γH2A.X	0.75	0.012
PSMα2	γH2A.X	0.77	0.0089
PSMα3	γH2A.X	0.82	0.0038
PSMα4	γH2A.X	0.7	0.024
δ-toxin	TotalRox	0.79	0.0062
PSMα1	TotalRox	0.88	0.00089
PSMα2	TotalRox	0.88	0.00088
PSMα3	TotalRox	0.9	0.00035
PSMα4	TotalRox	0.9	0.00033
δ-toxin	DAF2DA	0.7	0.024
PSMα1	DAF2DA	0.84	0.0026
PSMα2	DAF2DA	0.83	0.0028
PSMα3	DAF2DA	0.83	0.0031
PSMα4	DAF2DA	0.84	0.0022
δ-toxin	DCF	0.79	0.007
PSMα1	DCF	0.74	0.014
PSMα2	DCF	0.76	0.012
PSMα3	DCF	0.79	0.0065
PSMα4	DCF	0.78	0.0072

## Data Availability

The draft genome sequences of *S. aureus* isolates used in this study can be found in the NCBI Sequence Read Archive under the Bioproject ID PRJNA754839. Mass spectrometry proteomics data have been deposited to the ProteomeXchange Consortium via the PRIDE [58] partner repository with the dataset identifier PXD031622. All Skyline files of MRM experiments are published on PanoramaWeb [59] (https://panoramaweb.org/StaphylococcalPSM.url, accessed on 28 March 2022) and MRM raw data have been deposited to ProteomeXchange via PanoramaWeb [60] (http://proteomecentral.proteomexchange.org/cgi/GetDataset?ID=PXD031676, accessed on 28 March 2022).

## Supplementary Materials

The following supporting information can be downloaded at: https://www.mdpi.com/article/10.3390/cancers14092143/s1, Figure S1: *S. aureus* isolates selected for keratinocyte RNA-Seq and proteomics experiment are genetically and phenotypically distinct; Figure S2: *S. aureus* secretome-mediated intracellular-ROS signal in keratinocytes is dose- and time-dependent and can be negated by antioxidant N-acetyl cysteine, and *S. aureus* secretome causes human keratinocytes to produce nitric oxide; Figure S3: *S. aureus* secretome stimulates endogenous production of ROS by human keratinocytes. Figure S4: *S. aureus* secretome causes minor loss in keratinocyte viability that correlates with the magnitude of DNA damage. Figure S5: Exposure to *S. aureus* secretome increases frequency of oxidative DNA lesions in human keratinocytes; Figure S6: Phenol-soluble modulin concentration in staphylococcal secretomes; Figure S7: *S. aureus* culture density and secretome protein content do not correlate with secretome-induced DNA damage in human keratinocytes; Table S1: Differential expression analysis results; Table S2: Pathway enrichment analysis results; Table S3: PSM MRM transition list_complete; Table S4: PSM MRM transition list_final; Table S5: PSM MRM transition results
