# Supplementary material for: Skin Cancer-Associated S. aureus Strains Can Induce DNA Damage in Human Keratinocytes by Downregulating DNA Repair and Promoting Oxidative Stress
Source: Cancers (Basel). 2022 Apr 25;14(9):2143. doi: 10.3390/cancers14092143 (PMC9106025; doi:10.3390/cancers14092143)
Supplement: Supplementary file 1 [file cancers-14-02143-s001.zip › Figures S1-S7.pdf]

Supplementary Materials

Article

Skin cancer-associated *S. aureus* strains can induce DNA damage in human keratinocytes by downregulating DNA repair and promoting oxidative stress

Annika Krueger [1,2], Ahmed Mohamed [2], Cathryn Kolka [2], Thomas Stoll [2], Julian Zaugg [3], Richard Linedale [1], Mark Morrison [1], H. Peter Soyer [4,5], Philip Hugenholtz [3], Ian H. Frazer [1], and Michelle M. Hill [1,2,6,*]

[1] The University of Queensland Diamantina Institute, Faculty of Medicine, The University of Queensland, Translational Research Institute, Woolloongabba, Queensland, Australia
[2] QIMR Berghofer Medical Research Institute, Herston, Brisbane, Queensland, Australia
[3] The University of Queensland, School of Chemistry and Molecular Biosciences, Australian Centre for Ecogenomics, St Lucia, Queensland, Australia
[4] The University of Queensland Diamantina Institute, The University of Queensland, Dermatology Research Centre, Brisbane, Queensland, Australia
[5] Dermatology Department, Princess Alexandra Hospital, Brisbane, Queensland, Australia
[6] The University of Queensland Centre for Clinical Research, Faculty of Medicine, The University of Queensland, Herston, Queensland, Australia

Supplemental Figure S1

A

aureus isolate ID	Subject ID	Sample site	MLST ID	Strain name
SSA55	MS 2	AK	15	H451P
SSA57	MS 10	AK	1152	HT 2001 0706
SSA103	MS 14	SCC	1	H52
SSA110	MS 13	SCC	72	H309

B

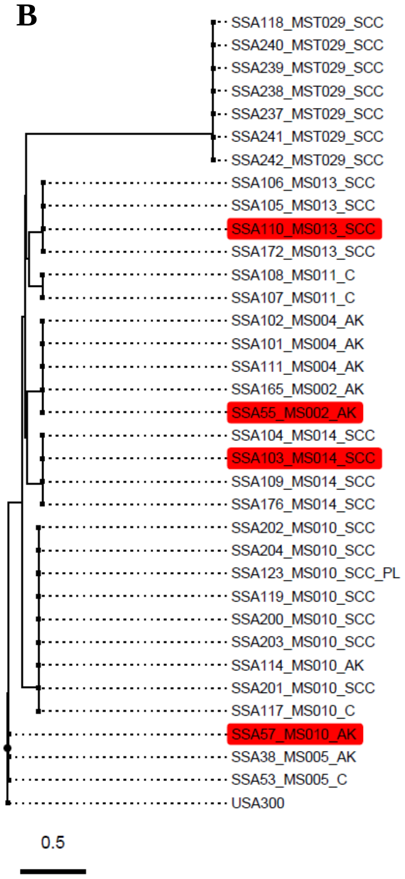

C

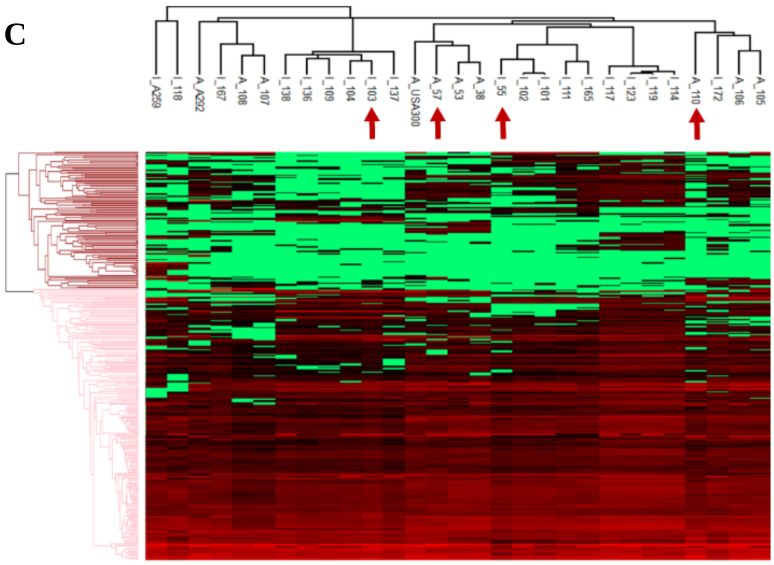

Figure S1. ***S. aureus*** **isolates selected for keratinocyte RNAseq and proteomics experiment are genetically and phenotypically distinct (A)** Strain origin and genetic background of the four S. aureus clinical isolates selected for the keratinocyte RNAseq and proteomics experiments. (**B**) Phylogenetic tree of S. aureus clinical isolates originating from eight different subjects from photo-damaged non-malignant skin (C), actinic keratosis lesions (AK), squamous cell carcinoma (SCC) and SCC perilesional skin controls (SCC_PL), in reference to type strain USA300. Indicated is the isolate ID (SSAxx), the subject the isolate was collected from (MS/Txx) and skin origin (C, AK or SCC). The four S. aureus isolates that were used in the keratinocyte RNAseq and proteomics experiment are highlighted in red and were genetically distinct. (**C**) Unsupervised hierarchical clustering of the protein/peptide profiles of different S. aureus strain secretomes as characterized by shotgun mass spectrometry. The four S. aureus isolates chosen for the keratinocyte RNAseq and proteomics experiment are highlighted with the red arrows. Proteomic analysis established that these four strains have diverse protein and peptide expression profiles relative to other clinical isolates available in our biobank.

Supplemental Figure S2

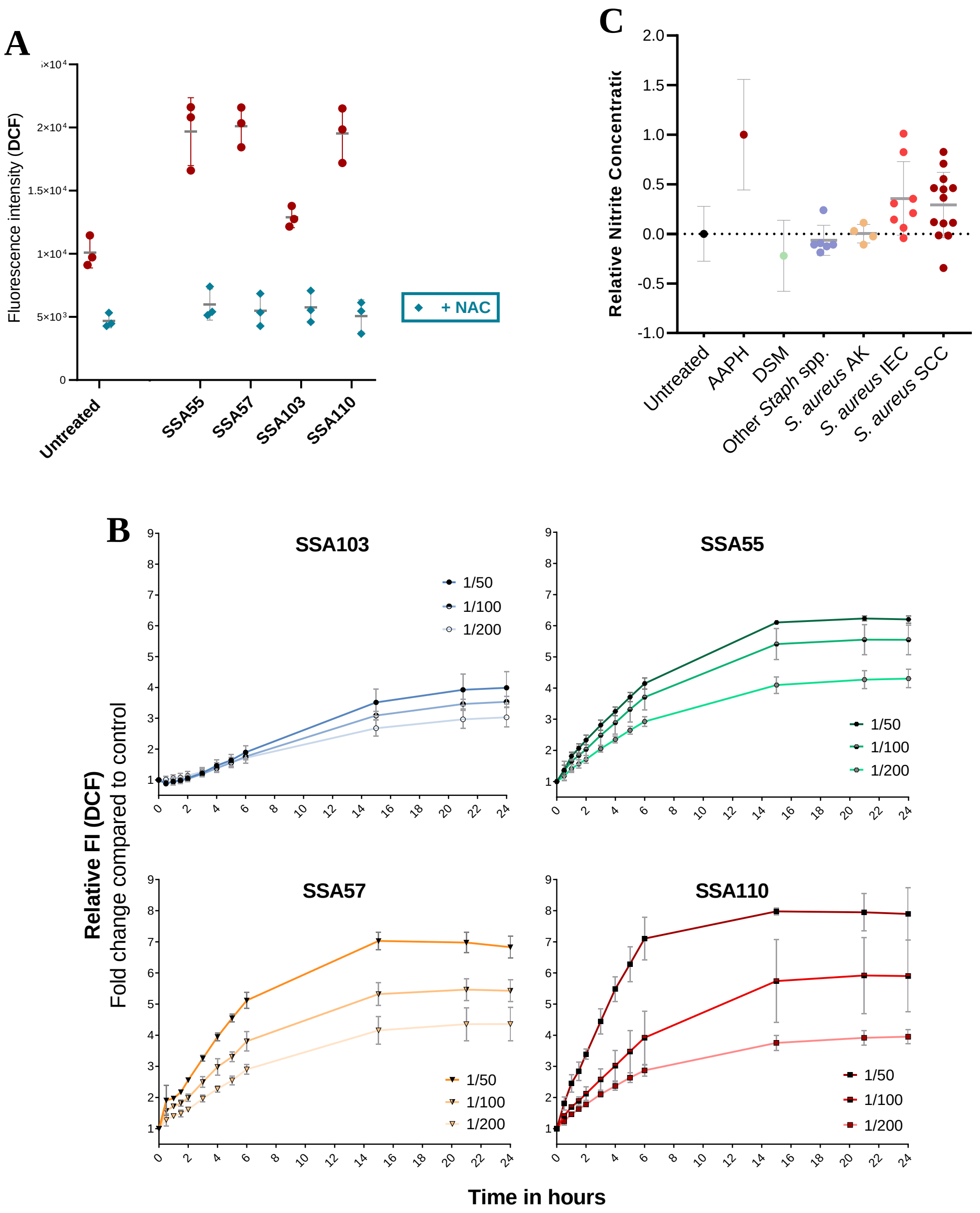

Figure S2. **S. aureus secretome-mediated intracellular-ROS signal in keratinocytes is dose- and time-dependent and can be negated by antioxidant N-acetyl cysteine, and S. aureus secretome causes human keratinocytes to produce nitric oxide (A)** Fluorescent intensity signal of DCF probe in primary human keratinocytes six hours post challenge with secretome from S. aureus clinical isolates +/- antioxidant N-acetyl cysteine (NAC, 2 mM). Indicated is the mean and SD of three technical replicates. NAC protects keratinocytes from S. aureus secretome-mediated ROS exposure. **(B)** ROS levels in keratinocytes monitored for 24 hours after addition of secretome from S. aureus clinical isolates (SSA55, SSA57, SSA103 and SSA110) at different concentrations: diluted 1:50, 1:100, or 1:200 in keratinocyte medium. ROS levels are quantified by measuring the fluorescent signal of ROS-oxidised probe (DCF) at λex/λem of 485/530 nm; displayed as fold change compared to untreated keratinocytes. Each data point indicates the mean and SD of three technical replicates. Keratinocyte intracellular ROS signal was time- and concentration-dependent. **(C)** Levels of nitrite, a stable breakdown product of nitric oxide (NO), in the culture supernatant of primary human keratinocytes after 24-hour challenge with secretome from S. aureus and other Staphylococcus species, as measured via colorimetric Griess assay. Each dot represents a different bacterial isolate and shows the grouped mean value from three independent experiments, each performed in technical triplicates.

Supplemental Figure S3

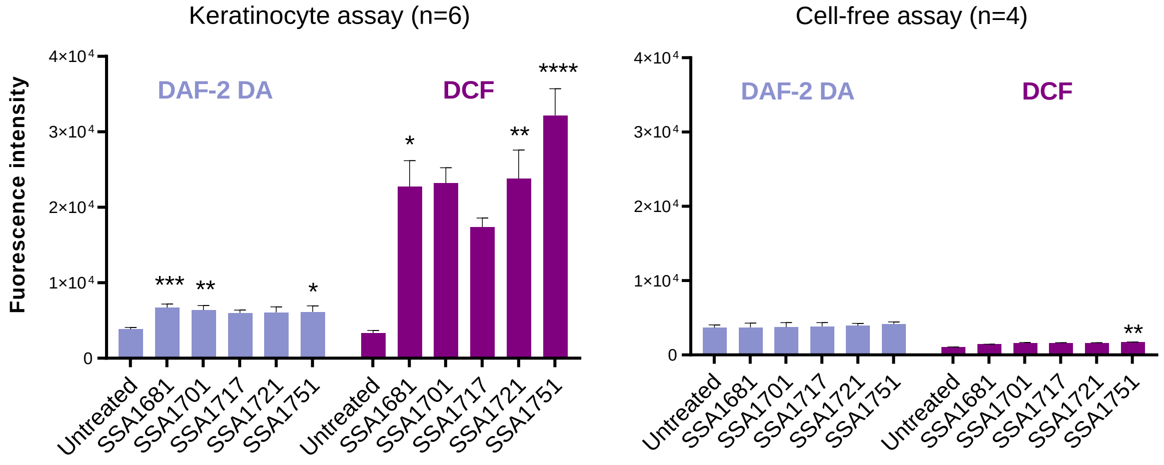

Figure S3. ***S. aureus*** **secretome stimulates endogenous production of ROS by human keratinocytes** Fluorescent intensity signal of ROS-sensitive DAF-2 DA and DCF-DA probes after 6-hour exposure to S. aureus secretome that was kept at 4°C for 12 days prior to experiment; either as intracellular signal within human keratinocytes (left graph, n=6 for each data point from two independent biological replicates; displayed as mean and SD) or directly in the bacterial secretome in a cell-free assay (right graph; n=4 for each data point; displayed as mean and SD). Significance determined between control and treatments for each data set via Dunn's multiple comparisons test. Keeping S. aureus secretome in the fridge for a prolonged time is sufficient for naturally containing ROS to dissipate as virtually no DAF-2 DA or DCF is observed in the cell-free assay. However, keratinocyte intracellular ROS signal to ROS-free S. aureus secretome remains strong, indicating that other more stable components of secretome can induce keratinocytes to produce ROS.

Supplemental Figure S4

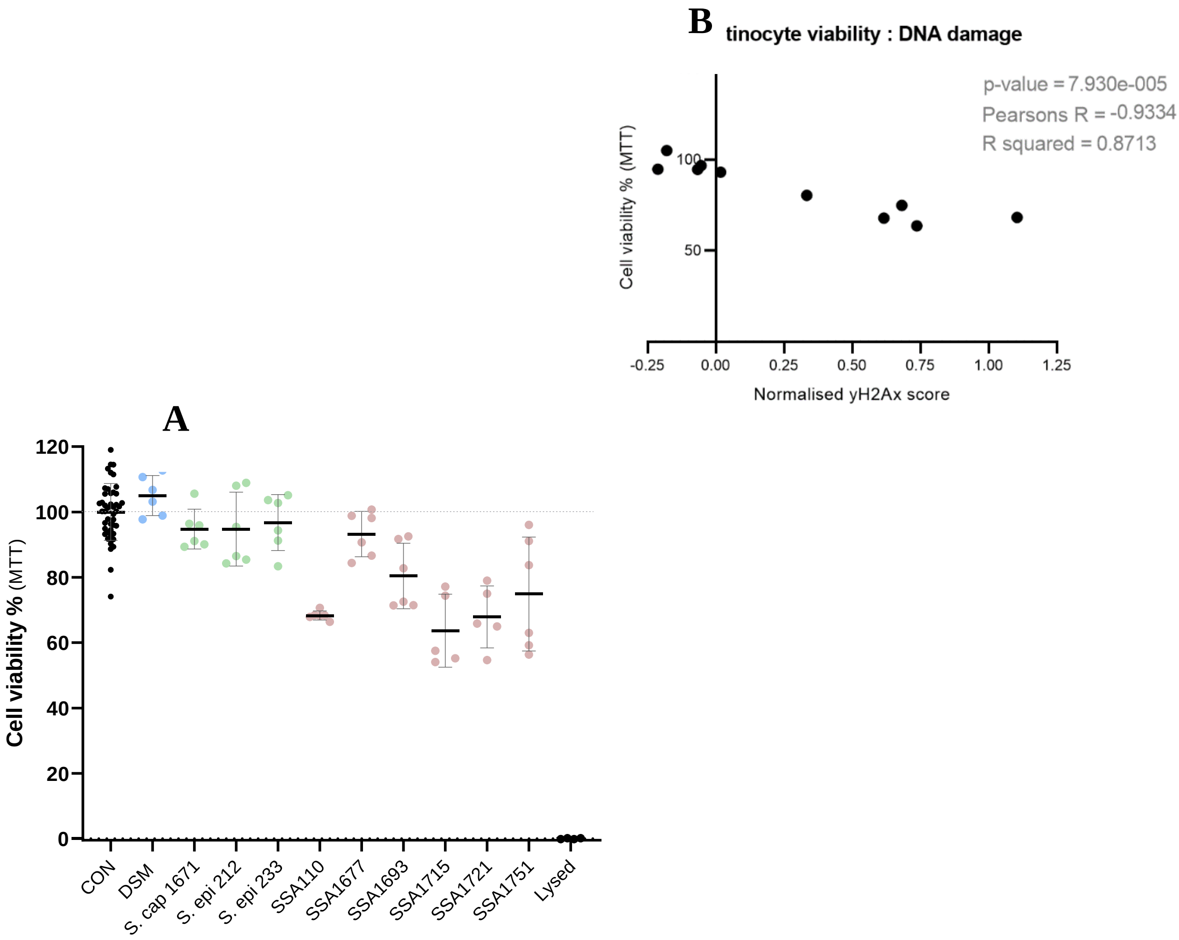

Figure S4. ***S. aureus*** **secretome causes minor loss in keratinocyte viability that correlates with the magnitude of DNA damage (A**) Cell activity measured via MTT assay indicating the viability of primary human keratinocytes after 24-hour treatment with secretomes from S. aureus and other Staphylococcus species that were tested in the γH2A.X DNA damage experiment (**Figure 5**). Values are normalised based on untreated keratinocytes (CON = 100%) and lysed cells (= 0%). Data from two independent biological replicates (total replicates: control n=45, treatments n=6). (**B**) Significant inverse correlation between secretome-induced DNA damage and keratinocyte viability (*p*-value = <0.0001; Pearson's R = -0.93).

Supplemental Figure S5

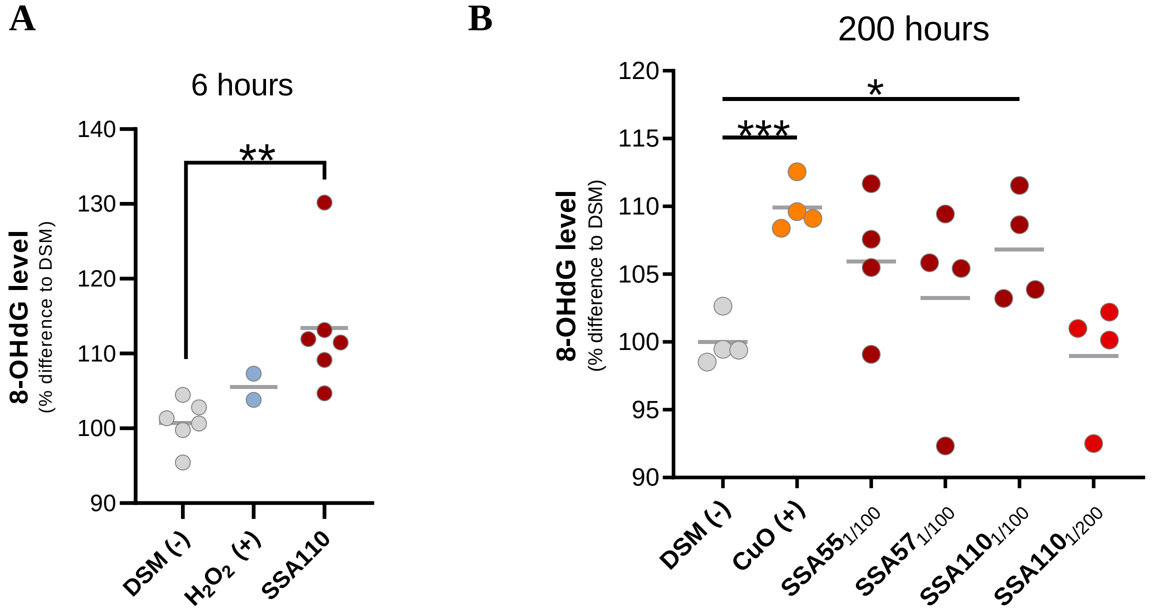

Figure S5. Exposure to *S. aureus* secretome increases frequency of oxidative DNA lesions in human keratinocytes (A) 8-OHdG levels in primary human keratinocytes (foreskin) in 3.5 µg digested DNA after short-term (6-hour) treatment with S. aureus SSA110 secretome diluted 1:100 in serum-free keratinocyte media. Bacterial growth medium DSM served as negative control and 200 µM hydrogen peroxide (H_2O_2) was used as positive control. Individual values with mean from three independent biological replicates, each performed in technical duplicates. Statistical significance determined between negative control DSM and SSA110 by unpaired t-tests. Secretome from S. aureus isolate SSA110 causes significantly higher levels of 8-OHdG compared to the negative control. (**B**) 8-OHdG levels in primary human keratinocytes (2x foreskin; 2x abdominal) in 2.5 µg digested DNA after long-term (200-hour) treatment with S. aureus secretomes diluted 1:100 in serum-free keratinocyte media; apart from one sample 1:200 as indicated. DSM served as negative control and copper oxide (CuO; 1 µg/ml) as positive control. Individual values with mean from four independent biological replicates. Long-term treatment with CuO and S. aureus secretome (1:100) appears to cause a small increase in frequency of 8-OHdG DNA lesions, although outliers are present. Statistical significance testing was performed between the negative control (DSM) and the remaining groups via unpaired t-tests.

Supplemental Figure S6

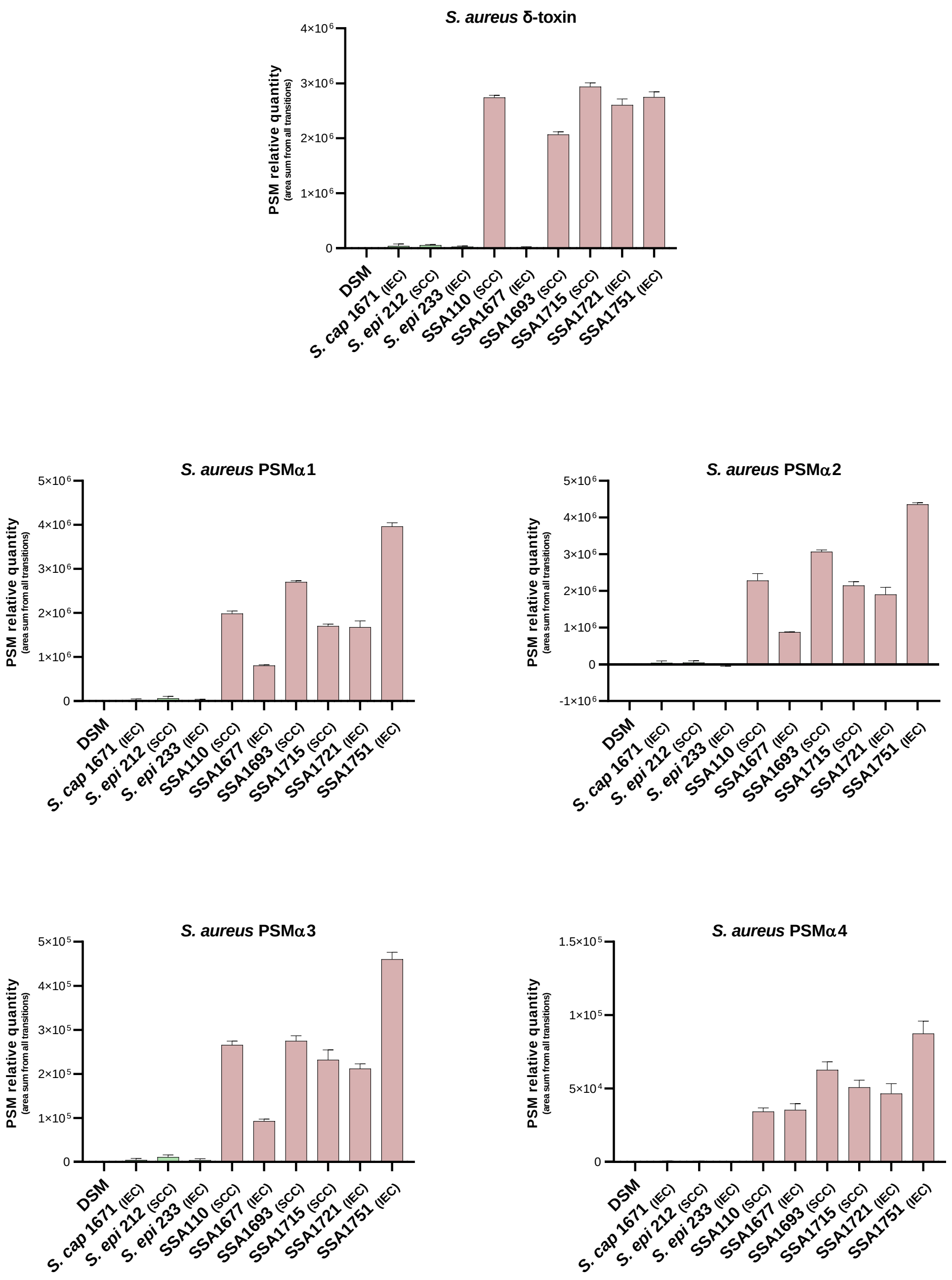

Figure S6. Phenol-soluble modulin concentration in staphylococcal secretomes Relative quantity of different PSM peptides in secretome samples of Staphylococcus isolates as determined by targeted mass spectrometry.

Supplemental Figure S7

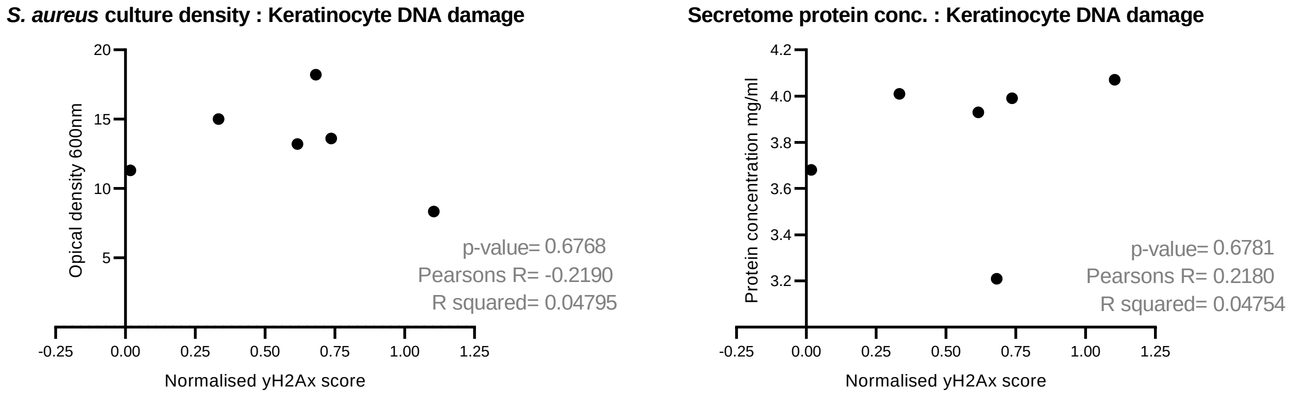

S. aureus isolate ID	Optical density of culture at harvest	Secretome protein conc. in mg/ml
SSA110	8.34	4.07
SSA1677	11.3	3.68
SSA1693	15	4.01
SSA1715	13.6	3.99
SSA1721	13.2	3.93
SSA1751	18.2	3.21

Figure S7. *S. aureus* culture density and secretome protein content do not correlate with secretome-induced DNA damage in human keratinocytes The table outlines the optical density of S. aureus culture at time of secretome collection and the protein concentration in secretome for the six S. aureus isolates that were tested for ability to cause DNA damage in cultured human keratinocytes. Pearson's correlation between overall protein quantity in secretome sample and its ability to cause DNA damage in human keratinocytes is non-significant (p=0.6781; right graph). Likewise, no significant correlation between the S. aureus culture density at time of secretome harvest and secretome-induced DNA damage (p = 0.6768; left graph).

Supplementary Table S1: ***Separate excel file Differential expression analysis results***

Supplementary Table S2: ***Separate excel file Pathway enrichment analysis results***

Supplementary Table S3: ***Separate excel file PSM MRM transition list_complete***

Supplementary Table S4: ***Separate excel file PSM MRM transition list_final***

Supplementary Table S5: ***Separate excel file PSM MRM transition result***
